# Celecoxib increases miR-222 while deterring aromatase-expressing breast tumor growth in mice

**DOI:** 10.1186/1471-2407-14-426

**Published:** 2014-06-12

**Authors:** Tsz Yan Wong, Fengjuan Li, Shu-mei Lin, Franky L Chan, Shiuan Chen, Lai K Leung

**Affiliations:** 1 Food and Nutritional Sciences Programme, School of Life Sciences, Faculty of Science, The Chinese University of Hong Kong, Rm.507C MMW Bldg, Shatin, Hong Kong; 2 Biochemistry Programme, School of Life Sciences, Faculty of Science, The Chinese University of Hong Kong, Shatin, Hong Kong; 3 School of Biomedical Sciences, Faculty of Medicine, The Chinese University of Hong Kong, Shatin, Hong Kong; 4 Division of Immunology, Beckman Research Institute of the City of Hope, Duarte, CA 91010, USA; 5 Department of Food Science, National Chiayi University, Chiayi City, Taiwan

**Keywords:** Celecoxib, Aspirin, Aromatase, miRNA

## Abstract

**Background:**

Breast cancer is one of the most deadly diseases in women. Inhibiting the synthesis of estrogen is effective in treating patients with estrogen-responsive breast cancer. Previous studies have demonstrated that use of cyclooxygenase (COX) inhibitors is associated with reduced breast cancer risk.

**Methods:**

In the present study, we employed an established mouse model for postmenopausal breast cancer to evaluate the potential mechanisms of the COX-2 inhibitor celecoxib. Aromatase-expressing MCF-7 cells were transplanted into ovariectomized athymic mice. The animals were given celecoxib at 1500 ppm or aspirin at 200 ppm by oral administration with androstenedione injection.

**Results:**

Our results showed that both COX inhibitors could suppress the cancer xenograft growth without changing the plasma estrogen level. Protein expression of ERα, COX-2, Cyclin A, and Bcl-xL were reduced in celecoxib-treated tumor samples, whereas only Bcl-xL expression was suppressed in those treated with aspirin. Among the breast cancer-related miRNAs, miR-222 expression was elevated in samples treated with celecoxib. Further studies in culture cells verified that the increase in miR-222 expression might contribute to ERα downregulation but not the growth deterrence of cells.

**Conclusion:**

Overall, this study suggested that both celecoxib and aspirin could prevent breast cancer growth by regulating proteins in the cell cycle and apoptosis without blocking estrogen synthesis. Besides, celecoxib might affect miR expression in an undesirable fashion.

## Background

Cyclooxygenase (COX) or prostaglandin G/H endoperoxide synthase is the enzyme responsible for converting arachidonic acid into prostaglandins [1]. Two isozymes of COX with differential expression patterns have been identified. COX-1 is constitutively expressed, and is involved in normal physiological functions, such as vascular homeostasis, platelet aggregation, gastric mucosa protection and maintenance of renal blood flow [2]. In contrast, COX-2 expression can be induced by cytokines and growth factors. This implies that COX-2 has a greater involvement in inflammation and cancer development than COX-1 [3]. Studies have shown that COX-2 is expressed in breast cancer tissues but not in normal breast tissues [4,5]. The concentrations of prostaglandin E_2_ (PGE_2_) in tumor and metastatic tissues are also higher than those in normal tissues [6]. The significance of COX-2 in breast carcinogenesis has also been described in different levels of research. Over-expressing COX-2 in mice promotes breast cancer development [7], whereas the administration of COX-2 inhibitor could prevent against breast carcinogenesis [8-10].

Aspirin is a non-steroidal anti-inflammatory drug (NSAID) that inhibits both COX-1 and COX-2. It is capable of deterring the growth of breast cancer cells [11]. Regular use of aspirin after breast cancer diagnosis improves survival [12]. In contrast, celecoxib is a new NSAID that specifically inhibits COX-2 and has drawn much attention for its anti-cancer properties. The COX-2 inhibitor reduces mammary tumor incidence induced by DMBA in rats [13]. It is also effective in blocking the growth of breast cancer xenografts in nude mice [14]. Celecoxib could evoke cell cycle arrest, anti-angiogenesis [15], and apoptotic cell death [16,17] in cancers. Although these NSAIDs appear to be chemopreventive, side effects like gastrointestinal tract bleeding [18] and cardiovascular toxicity [19] have been reported.

MicroRNAs (miRNAs) are small noncoding RNAs of about 22 nucleotides (nt) in length, and they can regulate gene expression at the post-transcriptional level. These single-stranded miRNAs bind to the 3′ untranslated region (3′ UTR) of target mRNAs, and cause translation blockage and/or mRNA degradation [20]. Studies have shown that miRNAs may regulate biological processes, like differentiation [21], cell growth and death [21], and tumorigenesis [22,23]. Many miRNAs are under-expressed in human tumors compared to normal tissues [24].

The objective of this study was to determine differential gene expression and other potential growth-suppressing mechanisms in breast tumorigenesis after celecoxib and aspirin treatment. We hypothesized that aromatase activity and miRNA regulation could be differentially inhibited by the two NSAIDs.

## Methods

Celecoxib was a gift from Pfizer Corp. Hong Kong Ltd. Aspirin was obtained from Sigma Chemical Co. (St. Louis, MO). Other chemicals were ordered from Sigma Chemical, if not stated.

### Cell culture

MCF-7 cells stably transfected with human *CYP19* (MCF-7aro) were prepared as previously described [25]. These cells were maintained in MEM medium (Invitrogen, Grand Island, NY) supplemented with 10% fetal bovine serum (Invitrogen Life Technology, Rockville, MD) and the selection antibiotic G418 (500 μg/ml, USB, Cleveland, OH). They were incubated at 37°C in 5% carbon dioxide and routinely sub-cultured when reaching 80% confluency.

### Part I. Animal experiment

This mouse model for postmenopausal breast carcinogenesis was described by Yue et al. [26]. Six-week old female athymic mice were acquired from the Animal Facility of Chinese University of Hong Kong. These mice were ovariectomized and allowed 3 weeks to recover, and were fed purified phytoestrogen-free AIN-93G diet. They were transplanted with MCF-7aro cells and randomly assigned into 4 regimens: control mice (Control), mice injected with androstenedione (AD), mice injected with androstenedione and treated with celecoxib (AD + celecoxib) and mice injected with androstenedione and treated with aspirin (AD + aspirin). The AD, AD + celecoxib and AD + aspirin mice received daily *s.c.* injections of androstenedione (0.1 mg dissolved in 0.1 ml 0.3% hydroxyl propyl cellulose). Control mice received the carrier solvent injection only. Celecoxib and aspirin were administered in the diet at 1500 ppm and 200 ppm, respectively. Before transplantation, MCF-7aro cells were maintained in a culture incubator as described above. The cells were trypsinized and suspended in matrigel matrix (BD Biosciences, San Jose CA) (10 mg/ml) at 3 × 10^7^ cells/ml. One hundred μl of cells were injected into the two flanks of the animal. This experiment was approved by Department of Health, the Governemnt of the Hong Kong SAR (Ref (07–164) in DH/ORHI/8/2/1 pt.9), and Animal Experimentation Ethics Committee of the Chinese University of Hong Kong (Ref. 13/023/GRF).

The body weight, tumor size and food intake were monitored weekly throughout the study. Tumor volumes were measured by an electronic caliper and estimated according to the formula: π/6 × length × width × height, where length, width, and height were the three orthogonal diameters of the tumors. At the end of the study, the mice were euthanized by cervical dislocation. Livers and uteri were weighed. Tumors and serum were collected and stored at -80°C until assayed.

#### ***Quantitative real time PCR assay***

The frozen tumor samples were pulverized in a Dounce homogenizer with liquid nitrogen. Total RNA was extracted from the sample using TRIzol reagent (Invitrogen, Carlsbad, CA, USA). The concentration and purity of RNA were determined by absorbance measured at 260/280 nm. First DNA strands were synthesized from 3 μg total RNA using 5× primers (Assay-on-Demand™, Applied Biosystems, Foster City, CA, USA) and MMLV reverse transcriptase (USB Corporation, Cleveland, OH, USA). Target fragments were quantified by DNA Engine Opticon II (MJ Research, Inc., Waltham, MA). Probes for amplification were obtained from Assay-on-Demand™, Applied Biosystems, i.e. the housekeeping U6 snRNA (Assay ID: 001973), has-miR-let-7c (Assay ID: 000379), has-miR-Let-7 g (Assay ID: 002282), has-miR-98 (Assay ID: 000577), has-miR-221 (Assay ID: 000524), has-miR-222 (Assay ID: 002276), has-miR-17-5P (Assay ID: 000393), has-miR-101 (Assay ID: 002253), has-miR-145 (Assay ID: 002278). We used the Real-time PCR Taqman Universal PCR Master Mix (Applied Biosystems) to set up the PCR reactions as described in the manual. Signals obtained from U6 were utilized for normalization, and relative gene expression were analyzed by using the 2^-ΔΔCT^ method [27].

For the determination of MYC and E2F2 RNA expression, we used oligo-dT primers for the first strand synthesis, SYBR green PCR Master Mix Reagent kit (Applied Biosystems) for the reaction setup, and GAPDH for normalization. The gene-amplification primer sequences were shown as below. By analyzing the dissociation curves and gel images, these primers did not produce non-specific amplifications. The relative gene expression was also determined by the 2^-ΔΔCT^ method (see Table 1).

**Table 1:** List of primers designed for RT-PCR quantitation

	**Forward primer sequence**	**Reverse primer sequence**
**MYC**	5′-TCT TCC AGA TAT CCT CGC TG-3′	5′-TAT GAC CTC GAC TAC GAC TCG-3′
**E2F2**	5′-TTA CAG TCA GAG GCC TGG CT-3′	5′-TTC TAA TAC TCA TCC CTG TTT TTC C-3′
**GAPDH**	5′-GAG TCA ACG GAT TTG GTC GT-3′	5′-GAT CTC GCT CCT GGA AGA TG-3′

#### ***Immunoblot of proteins extracted from MCF-7aro tumors***

The frozen samples were pulverized in a Dounce homogenizer with added liquid nitrogen. The pulverized samples were then sonicated in lysis buffer (PBS, 1% NP40, 0.5% sodium deoxycholate, 0.1% SDS, 40 mg/L PMSF, 0.5 mg/L aprotinin, 0.5 mg/L leupeptin, 1.1 mmol/L EDTA and 0.7 mg/L pepstatin) with a cell disruptor (Branson Ultrasonics Corp., Danbury, CT, U.S.A.) on ice for 30 s for protein extraction. Thirty μg of protein extract were separated on 10% SDS-PAGE and transferred onto an Immobilon PVDF membrane (Millipore, Bedford, MA). Anti-ERα, anti-COX-2, anti-CDK4, Cyclin A, E, anti-Bcl-xL, Bcl-2, Bax, Bak (Santa Cruz Biotechnology, Santa Cruz, CA, USA), and anti-β-actin primary (Sigma Chem) and secondary antibodies conjugated with horseradish peroxidase (Santa Cruz Biotechnology) were used for protein detection. The targeted proteins were visualized by autoradiography on a Biomax (Kodak®) film. The images were scanned and analyzed for optical density by using the computer software ImageJ (National Institute of Mental Health, Bethesda MD, USA).

#### ***Serum estradiol determination***

Serum estradiol concentration was measured by using ELISA kits from Cayman Chemical Company (Ann Arbor, MI). The samples were added into a 96-well plate coated with antibody raised against estradiol. After incubating with the tracer and developing at room temperature, the absorbance was quantified using a microplate reader (FluroStar®, BMG Labtechnologies GmBH, Offenburg, Germany). The amount of estradiol could be read against a standard curve constructed with the hormone provided in the kit.

### Part II. *In vitro* experiments

#### ***CYP19 enzyme inhibition assay***

Two pmol recombinant aromatase protein (human CYP19 Supersomes®, BD Gentest, Woburn, MA) was incubated with celecoxib or aspirin in the substrate-containing assay buffer (25 nM-[1β-^3^H(N)] androst-4-ene-3,17-dione (NET-926; Perkin-Elmer Life and Analytical Sciences, Boston, MA), 3 · 3 mM-MgCl_2_, 100 mM-KH_2_PO_4_ (pH 7 · 4)). The reaction was initiated by adding 1.3 mM-NADPH and incubated at 37°C for 15 min. An aliquot of the medium was mixed with chloroform and centrifuged at 10,000 g for 10 min at 4°C. The aqueous phase was transferred into a tube containing 500 μl of 5% activated charcoal. An aliquot of the supernatant was removed for scintillation counting after incubating for 30 min.

#### ***Verification of expression pattern in culture cells***

MCF-7aro cells were seeded in culture dishes at 5 × 10^2^ cells/mm^2^, and allowed to settle for 1 day before treatment began. They were co-treated with androstenedione and various concentrations of aspirin or celecoxib for 1–3 days with DMSO as the carrier solvent. The final concentration of solvent was 0.1% (vol/vol). Total protein or RNA was extracted and analyzed.

#### ***Relationship between the differentially expressed genes and miR-222/-98***

MCF-7aro cells were cultured in OptiMEM (Invitrogen Life Technology) and transfected with miR-222 or miR-98 mimics (Invitogen Life Technology) in Lipofectamine 2000 (Invitrogen Life Technology). Six hr after the transfection, the culture medium was replaced with RPMI (phenol red free) supplemented with 10nM androstenedione and 5% charcoal-dextran treated fetal bovine serum (Biotechnics Research, CA USA). Total protein or RNA was extracted 72 hr after the medium change. MTT assays were also performed in separate experiments to investigate the effect on cell growth.

### Statistical methods

The software package Prism® 5.0 (GraphPad Software, Inc., CA, USA) was employed for statistical analysis. For multiple group analysis, the data were analyzed by *One-way ANOVA* followed by *Tukey’s Multiple Comparison* if significant differences (P < 0.5) were observed.

## Results

### Celecoxib and aspirin treatment had no effect on mouse body weight and liver weight

**Figure 1:**
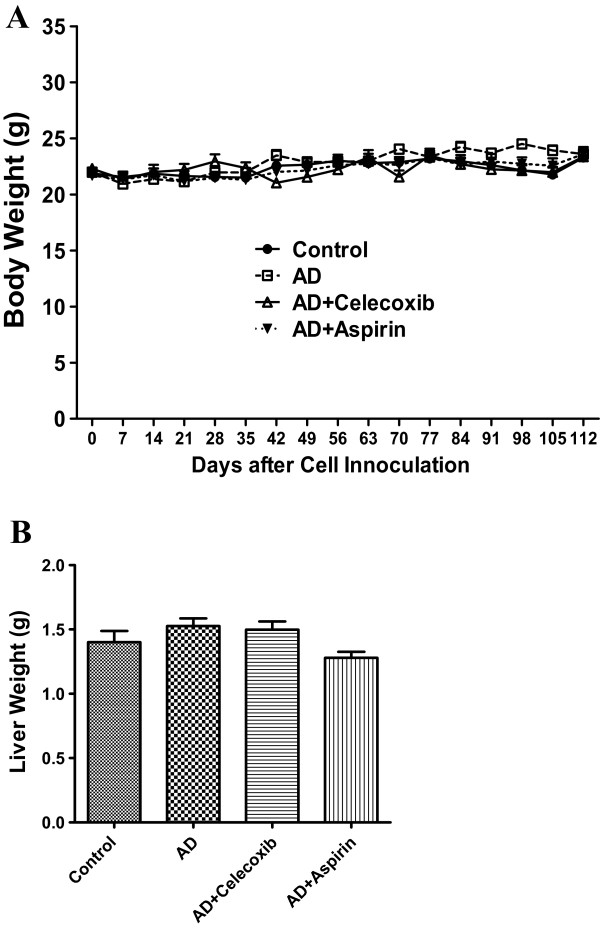
**Celexoxib or aspirin treatment had no effect on body weight and liver weight.** Mice were inoculated with MCF-7aro cells and treated with celecoxib, aspirin and androstenedione. Their body weight **(A)** was monitored from the second week after inoculation and liver weight **(B)** was measured at the end of experiment. Groups labeled as AD, AD+celecoxib, and AD+aspirin are the mice treated with androstenedione, androstenedione and celecoxib, and androstenedione and aspirin, respectively. Values are means ± SEMs, n=6 to 8. The data was analyzed by One-way ANOVA, followed by Tukey’s Multiple Comparison test when *P*<0.05.

The body weights of all mice gradually increased and no significant differences were observed among the groups at any given time point (Figure 1A). Similar to the body weights, no significant differences were observed in liver weights at euthanasia (Figure 1B). The drug treatment appeared to be within the tolerable limit.

### Effect of celecoxib and aspirin on xenograft growth

**Figure 2:**
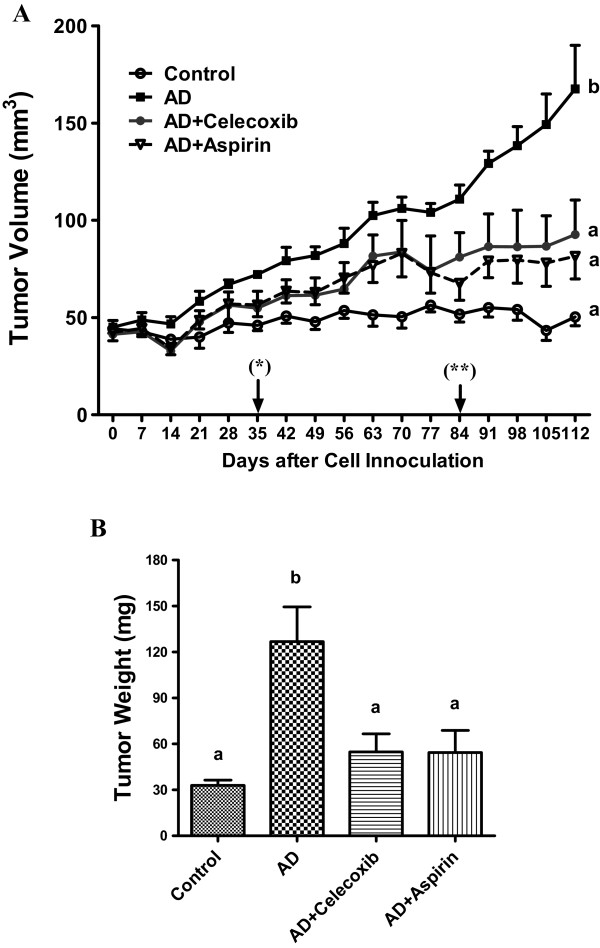
**Effect of celecoxib and aspirin on the growth of MCF-7aro xenograft.** Mice were inoculated with MCF-7aro cells at 2 sites per mouse after ovariectomy. They were injected subcutaneously with androstenedione every day starting on the next day after inoculation. Tumor volumes **(A)** were estimated once a week from Day 7 after inoculation. Day labeled as (*) was the time when AD Group significantly deviated from Control Group sacrifice. (**) was the day when tumor volumes of AD+Celecoxib and AD+Aspirin started deviated from that of AD. The tumor weight was measured at the day of sacrifice **(B)**. Groups labeled as AD, AD+celecoxib, and AD+aspirin are the mice treated with androstenedione, androstenedione and celecoxib, and androstenedione and aspirin, respectively. Values are means ± SEM, n=6 to 8 (2 inoculation sites combined as one data point). The data was analyzed by One-way ANOVA, followed by Tukey’s Multiple Comparison test when *P*<0.05. Means labeled with different letters are significantly different (*order: b>a*).

Accelerated tumor growth was recorded in mice treated with androstenedione (AD) as compared with that of Control. Tumor volume in AD mice became significantly (P < 0.05) different from that in Control starting from Day 35 until sacrifice. Beginning from Day 84 until euthanasia, tumor volumes in the treatment groups AD + celecoxib and AD + aspirin were significantly (P < 0.05) smaller than those in AD mice (Figure 2A). The tumor weights measured at euthanasia were consistent with the tumor volume data (Figure 2B).

### Celecoxib decreased ERα expression without affecting plasma estradiol concentration and uterine weight

**Figure 3:**
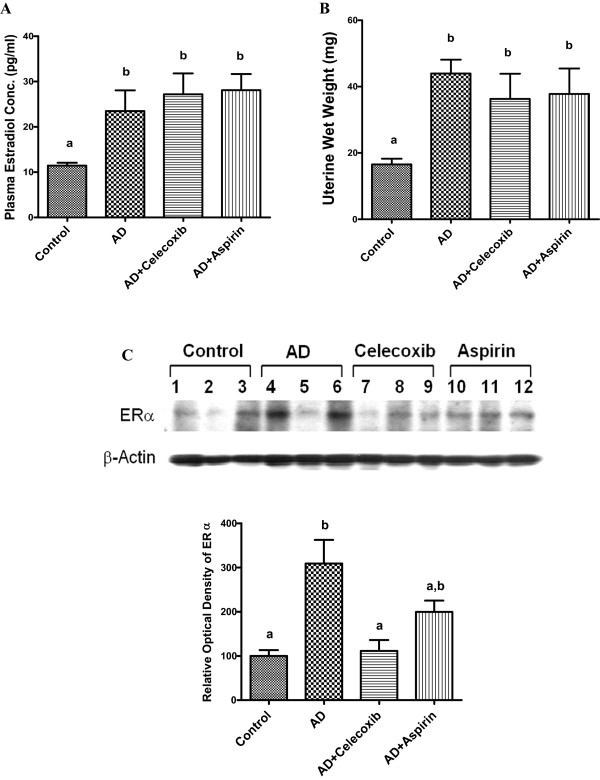
**Effect of celecoxib and aspirin on plasma estradiol concentration, uterine weight and ERα expression.** Blood was drawn from the animals at sacrifice. Serum estradiol concentration was quantified by ELISA **(A)**. Sample labeled as **AD** is androstenedione. Uterine of the experimental animals were dissected and weighed at sacrifice as shown in **B**. Western blot analysis of ERα in xenograft samples was analyzed by immunoblotting **(C)** and the optical density measurements were shown in the lower panel. Groups labeled as AD, AD+celecoxib, and AD+aspirin are the mice treated with androstenedione, androstenedione and celecoxib, and androstenedione and aspirin, respectively. Values are means ± SEM, n=6 to 8.The data was analyzed by One-way ANOVA, followed by Tukey’s Multiple Comparison test when *P*<0.05. Means labeled with different letter are significantly different (*order: b>a*).

All mice received androstenedione had higher plasma estradiol concentrations than those in Control mice. No reduction in estradiol was observed in mice treated with celecoxib or aspirin (Figure 3A). AD mice exhibited a two-fold increase in uterine wet weight over that of Control. Treatment with celecoxib or aspirin did not significantly change the androstenedione-induced uterine weight (Figure 3B). The uterine weight data were consistent with the plasma estrogen concentrations. The androstenedione-induced expression of ERα, on the other hand, was reversed by celecoxib administration (Figure 3C).

### Expression of COX-2, cell cycle and apoptosis-related proteins in tumors

**Figure 4:**
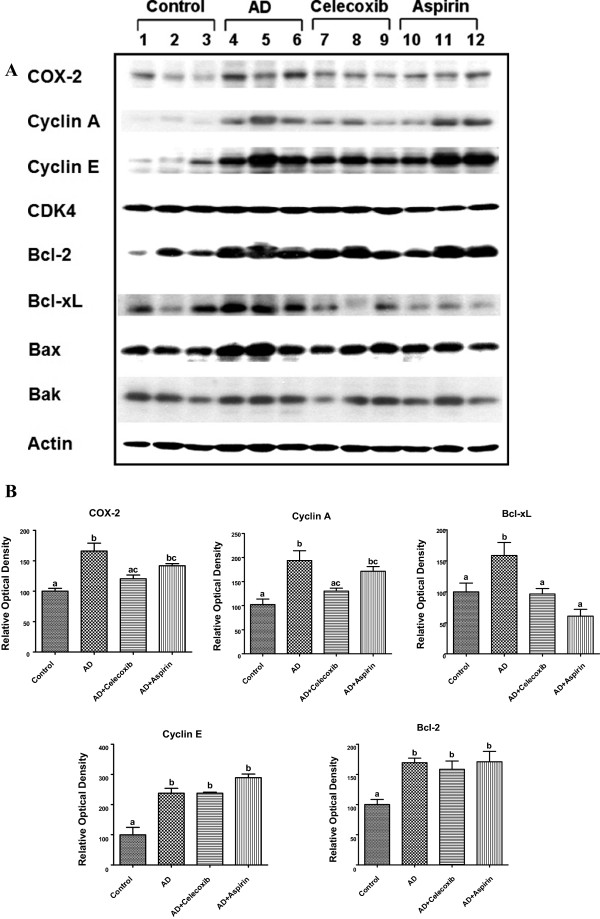
**Protein expression of COX-2 and genes related to cell cycle and apoptosis.** COX-2, cell cycle and apoptosis regulatory proteins were determined by western blot analysis, and the image is shown in **A**. The corresponding optical density reading is shown in **B**. Groups labeled as AD, AD+celecoxib, and AD+aspirin are the mice treated with androstenedione, androstenedione and celecoxib, and androstenedione and aspirin, respectively. Values are means ± SEM, n=3 to 5. The data was analyzed by One-way ANOVA, followed by Tukey’s Multiple Comparison test when *P*<0.05. Means labeled with different letter are significantly different (*order: b>a*).

Since both celecoxib and aspirin inhibited tumor growth in the animal model, we examined some proteins that are important in regulating cell growth. COX-2, Cyclin A & E, Bcl-2, and Bcl-xL were higher in AD tumor samples than those in the Control as shown in Figure 4. Both NSAIDs counteracted the androstenedione-induced Bcl-xL, while celecoxib could also neutralize the induced expression of COX-2 and Cyclin A. Other proteins (CDK4, Bax, and Bak) were not different among all treatment groups.

### MiRNA expression in tumors

**Figure 5:**
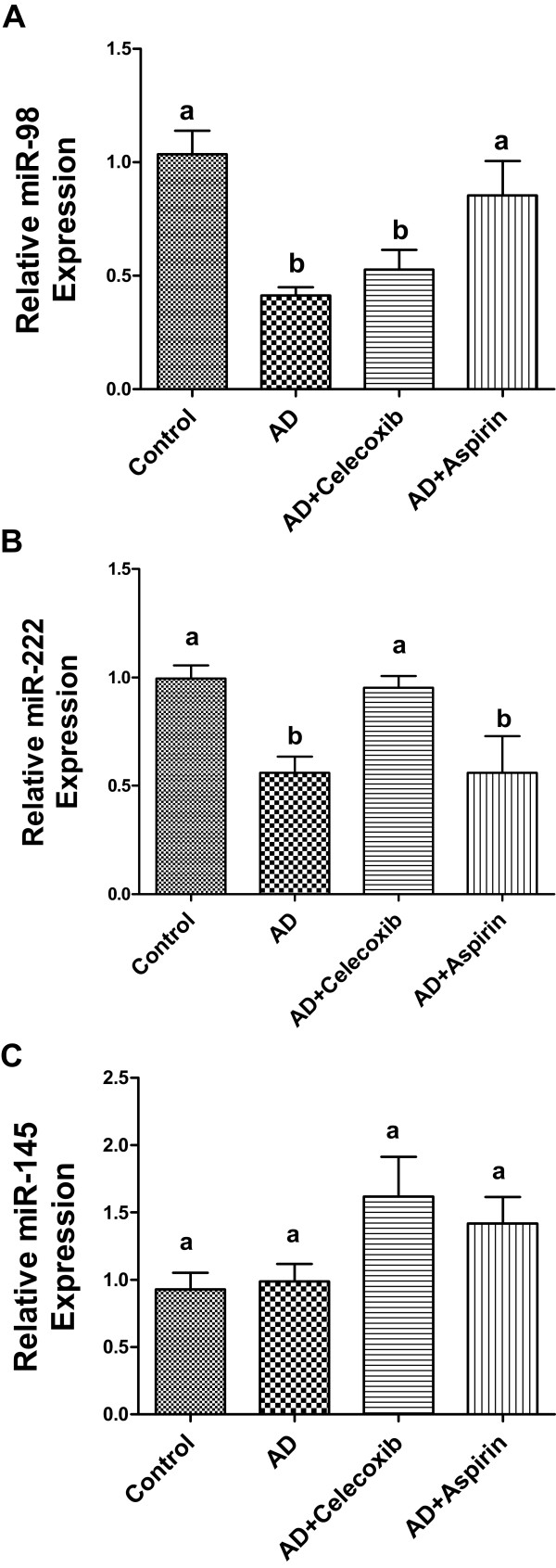
**MiRNA expression in tumors in mice treated with celecoxib and aspirin.** Total mRNA was extracted from tumors and miRNA expression of miR-98 **(A)**, 222 **(B)**, 145 **(C)** was quantified by real-time PCR. Groups labeled as AD, AD+celecoxib, and AD+aspirin are the mice treated with androstenedione, androstenedione and celecoxib, and androstenedione and aspirin, respectively. Values are means ± SEM, n = 6. The data was analyzed by One-way ANOVA, followed by Tukey’s Multiple Comparison test when *P*<0.05. Means labeled with different letters are significantly different (*order: a>b*).

Expression of breast cancer-associated miRNAs, including miR-let-7c, miR-let-7 g, miR-98, miR-221, miR-222, miR-101, miR-145 and miR-17-5p, in the xenografts was also measured. Among these, miR-98 (Figure 5A) and miR-222 (Figure 5B) were downregulated in AD mice. Aspirin and celecoxib could reverse the suppression of miR-98 and miR-222, respectively. Although the expression of miR-145 was not significantly changed among all groups, celecoxib tended to increase its level (Figure 5C). No significant changes were found in the expression of the other miRNAs (data not shown).

### MYC and E2F2 mRNA expression in tumors

**Figure 6:**
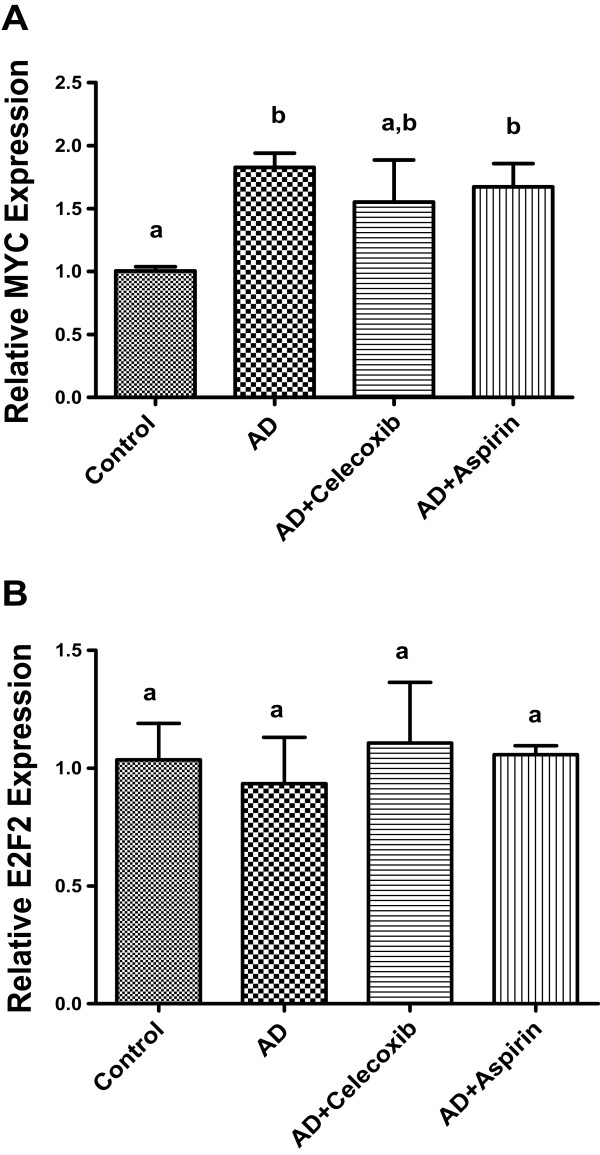
**The effect of drug treatment on c-Myc and E2F2 in tumors.** Total mRNA was extracted from tumors and mRNA expression level of c-Myc **(A)** and E2F2 **(B)** was quantified by real-time PCR. Groups labeled as AD, AD+celecoxib, and AD+aspirin are the mice treated with androstenedione, androstenedione and celecoxib, and androstenedione and aspirin, respectively. Values are means ± SEM, n = 6.The data was analyzed by One-way ANOVA, followed by Tukey’s Multiple Comparison test when *P*<0.05. Means labeled with different letter are significantly different (*order: b>a*).

A recent study has documented that MYC and E2F2 can be regulated by miR-98 [28]. The mRNA expression of MYC and E2F2 was examined as a follow-up study to the differential expression of miR-98 as shown above. Our results showed that the mRNA expression of MYC could be induced by androstenedione, but celecoxib and aspirin treatment had no counteracting effect on the expression (Figure 6A). No differences were found in the mRNA expression of E2F2 (Figure 6B).

### Celecoxib and aspirin were not aromatase inhibitors

Celecoxib (1–10 μM) and aspirin (1–1000 μM) had no inhibition on the aromatase activity of CYP19 recombinant protein (data not shown). These results were consistent with the null effect on plasma estrogen in mice.

### Verification of protein expression in the cell culture system

**Figure 7:**
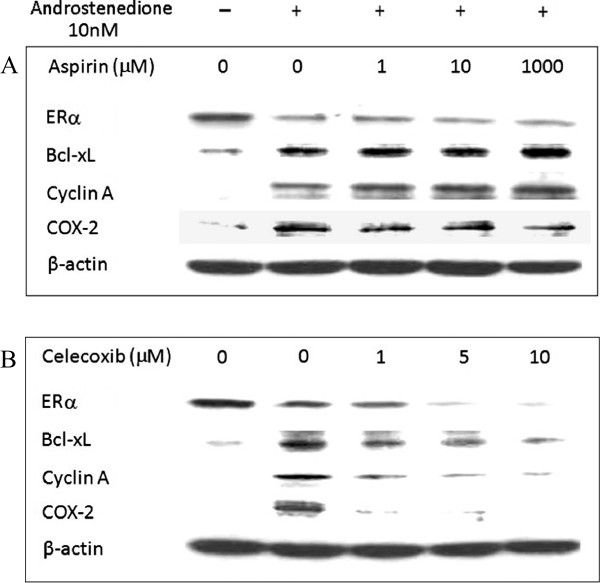
**Verification of protein expression in the cell culture system.** Western blot analysis was performed on a cell culture system. MCF-7aro cells were cultured and treated with 10 nM androstenedione and aspirin or celecoxib for 72 hr. Protein was extracted from the cells. The upper panel **(A**) is the image for aspirin-treated samples and celecoxib-treated samples are shown in the lower panel **(B)**.

Immunoblot results indicated that 10 nM androstenedione elevated the protein levels of Bcl-xL, Cyclin A, and Cox-2 and suppressed that of ERα. Celecoxib further suppressed ERα, and reduced the steroid-induced Bcl-xL, Cyclin A, and Cox-2 levels in the cultured MCF-7aro cells in a dose-dependent manner (Figure 7B). In contrast, aspirin did not affect any of those proteins in the culture system (Figure 7A). The results for celecoxib were consistent to those in the animal study.

### MiR-222 expression was induced by celecoxib in MCF-7aro cells *in vitro*

**Figure 8:**
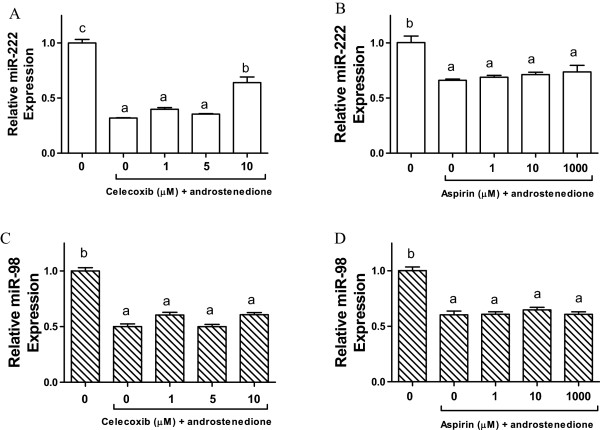
**MiR-98 and miR-222 expression in MCF-7aro cells treated with aspirin and celecoxib.** Cells are treated with androstenedione and celecoxib, and androstenedione and aspirin. Total mRNA was extracted from cells and miRNA expression of miR-98 and 222 was quantified by real-time PCR. MiR-222 expression of celecoxib and aspirin are shown in **A** &**B**, while **C** &**D** are the miR-98 expression of celecoxib and aspirin, respectively. Values are means ± SEM, n = 3. The data was analyzed by One-way ANOVA, followed by Tukey’s Multiple Comparison test when *P*<0.05. Means labeled with different letters are significantly different (*order: c>b>a*).

By using real-time PCR assay, we determined the expression of miR-222/-98 in cells treated with celecoxib and aspirin. Compared to the control, androgen administration suppressed the expression of miR-222 and miR-98. Ten μM celecoxib significantly (P < 0.05) reversed the suppression of miR-222 (Figure 8A), whereas no significant changes were observed for miR-98 (Figure 8C) or those cultures treated with aspirin (Figure 8B & D).

### MiR-222 could be a factor for ERα suppression

**Figure 9:**
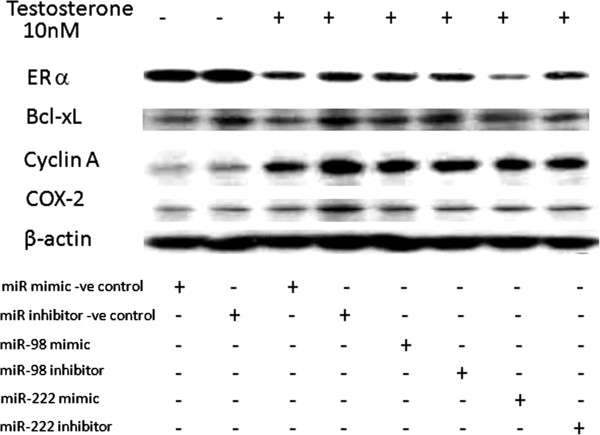
**Protein expression in miR-98 and miR-222 over-expressed MCF-7aro cells.** Cells are treated with androstenedione and transfected with miR-98 or miR-222. Protein was extracted from cells and expression of ERa, Bcl-xL, Cyclin A, and Cox-2 was quantified by western blot. The image represents one of two blots with similar results.

In order to investigate the connection between miR-222/-98 and the protein expression profile, MCF-7aro cells were transfected with mimic miR-222 and miR-98. Androstenedione could reduce ERα. The miR-222 mimic further lowered the ERα protein, while other proteins tested were not affected. MiR-98 had no effect on any of the proteins (Figure 9). The administration of miR-222 inhibitor did not change ERα expression compared with that of miR-222 inhibitor –ve control. The low baseline level of miR-222 could be the contributing factor. MTT assays were also performed in these cultures. No significant difference in cell growth was observed in cells transfected with miR-222 or -98 mimic after 72-h incubation (data not shown).

## Discussion

Previous studies have demonstrated that celecoxib at high concentrations can suppress aromatase activity [29] and reduce estradiol amount [30] in the cultured breast cancer cells SK-BR-3 and MCF-7/Cox-2 clone. In the present study, we could not validate the celecoxib’s inhibition on aromatase activity or expression in MCF-7 cells as high as 10 μM (data not shown). Furthermore, the Cox-2 inhibitor was also not effective in lowering estradiol concentration in an aromatase-expressing breast xenograft model. After all, neither celecoxib nor aspirin were suppressors to aromatase at any levels.

Overexpression of cyclins has been observed in breast cancer [31-33]. In contrast, celecoxib and aspirin inhibit cell cycle progression through G1 phase arrest in colon cancer cells [34,35]. In the present study, celecoxib but not aspirin reduced the protein levels of Cyclin A. Since the cyclin suppression is consistent with the condition required for G-1 phase arrest, the COX-2 inhibitor might block the cells from entering the S phase.

Apoptosis is a crucial process in the treatment of cancer. COX-2 promotes resistance against apoptosis by altering the levels of pro- and anti-apoptotic proteins [36,37]. Celecoxib induces apoptosis in breast cancer cells by differential regulation of Bcl-2 and Bax [38]. Aspirin is also able to induce apoptosis by down-regulating Bcl-2 protein expression in colon cancer cells and human gastric epithelial cells [39,40]. Rather than reducing Bcl-2, both celecoxib and aspirin decreased Bcl-xL in the present study.

Dysregulation of miRNAs has been demonstrated in breast carcinogenesis, and their involvement in cancer initiation and progression has been suggested [41,42]. MiR-98 may interact with and reduce the expression of *CYP19 *[43], c-Myc and E2F2 [28] in cells. Increased miR-222 species is associated with drug resistance and estrogen-independent growth [44]. MiR-145, on the other hand, is a tumor suppressor gene and is down-regulated in MCF-7 cells [45]. Over-expressing miR-145 in breast cancer cells suppresses the cell growth and induces apoptosis through downregulating ERα and Rhotekin expression [46,47]. In addition, miR-145 may also block the expression of Fli-1 and Bcl-2 in colon cancer cells [48]. Our study indicated that androstenedione suppressed miR -98 and -222, and aspirin and celecoxib reversed the expression in the tumors, respectively. The null result of miR-98 expression in cultures after aspirin treatment was inconsistent with the animal study data. Aspirin could act indirect in controlling miR-98. On the other hand, miR-222 was consistently upregulated by celecoxib administration in both *in vivo* and *in vitro* systems. The interrelationship between miR-222 and ERα in the current study was not determined. The induction of miR-222 expression might reduce ERα expression [49], or it could also be a direct result from downregulation of ERα [50].

## Conclusion

In summary, both COX inhibitors suppressed breast tumor growth. However, celecoxib might also upregulate the undesirable miR-222.

## Competing interests

The authors declare that no competing interests.

## Authors’ contributions

FL mostly performed the *in vivo* experiments, while TYW performed most *in vitro* experiments. SL participated in both *in vivo* and *in vitro* experiments of this study. FL & TYW also performed the statistical analysis. FC & SC constructed the transfection plasmid and prepared the stable cell line MCF-7aro. LKL designed and co-ordinated this study, and drafted the manuscript. All authors read and approved the final manuscript.

## Pre-publication history

The pre-publication history for this paper can be accessed here:

http://www.biomedcentral.com/1471-2407/14/426/prepub
